# New Interest in Wild Forest Products in Europe as an Expression of Biocultural Dynamics

**DOI:** 10.1007/s10745-017-9949-7

**Published:** 2017-10-25

**Authors:** K. F. Wiersum

**Affiliations:** Forest and Nature Conservation Policy group, Wageningen University and Research, Wageningen, The Netherlands

**Keywords:** Bio-cultural dynamics, Co-evolution, Domestication, Re-wilding, Non-wood forest products, Socio-ecological systems, The Netherlands

## Abstract

In Europe, interest in wild forest products is increasing. Such products may be interpreted in a biological sense as deriving from autonomously growing forest species or in a biocultural sense as reflecting dynamics in human living with biodiversity through re-wilding of earlier domesticated species. In this article I elaborate the idea that the new interests reflect biocultural dynamics. First, I identify these dynamics as involving both domestication and re-wilding and characterize these processes as involving biological, environmental, and cultural dimensions. Next, I present a comparative review of two approaches to re-wilding forest production in the Netherlands: meat production from new types of natural grazing systems, and food production from plants re-introduced to the wild. The first approach is based on the stimulation of naturally occurring ecological processes and the second on the stimulation of new forms of experiencing bio-cultural heritage. The examples demonstrate that the new interests in wild forest products involve both a return to earlier stages of domestication in an ecological sense and a new phase of acculturation to evolving socio-cultural conditions.

## Introduction

Since the 1990s, the role and importance of non-wood forest products (NWFPs) in tropical countries has received considerable attention (Shackleton *et al.* 2011). In analysing the scope for NWFP production initially much attention was given to its potential to contribute towards decreasing deforestation and improving forest conservation as well as contributing to livelihood improvement (Sunderlin *et al.* 2005; Kusters *et al.* 2006). Within this context much attention was given to the collection of wild products from natural forests. Gradually, it was recognised that the scope for NWFP production could be improved by considering NWFP production not just from natural forests but also from domesticated forests (Belcher *et al.* 2005; Ros-Tonen and Wiersum 2005). Such domestication of NWFPs has a long history in Europe. Many original NWFP species were taken out of the forests to be cultivated in specialized production systems such as fruit orchards or horticultural enterprises. Currently, in Europe only a few valuable NWFPs such as resin and cork are still considered forest products. Recently, however, new interest in wild non-timber forest products is emerging for culinary and other experiential purposes (Emery *et al.* 2006; Łuczaj *et al.* 2012; Schulp *et al.* 2014; Reyes-Garcia *et al.* 2015); these products are often collected as part of recreational activities (Emery *et al.* 2006; Stryamets *et al.* 2015; Wolfslehner *et al.* forthcoming). These NWFPs are not primarily serving basic livelihood needs by contributing to physiological and safety needs, but rather contribute to wellbeing in the sense of self-actualization (Kusel 2001; Wiersum *et al.* 2004). In view of Europe’s history of domesticating valuable NWFPs the question arises how these new cultural interests in wild forest products can best be understood in the context of human–nature relations.

Recently, the concept of biocultural diversity was identified to reflect the interaction between biodiversity and cultural diversity and their co-evolution in complex socio-ecological systems (Posey 1999; Persic and Martin 2008; Pretty *et al.* 2009). The interaction between people and biodiversity may take several forms ranging from linguistic expressions and spiritual beliefs to culturally specific practices for material and cultural use of biodiversity. The nature and dynamics of biocultural diversity have especially been explored in tropical countries (Posey 1999; Maffi and Woodley 2010). In these studies the concept has predominantly been applied to represent the nature-culture interactions of indigenous peoples and traditional societies, with much attention to documenting traditional biocultural practices. In this context several studies have identified the loss of such historic biodiversity due to modernisation (Pilgrim *et al.* 2008; Pilgrim and Pretty 2010; Rapport and Maffi 2010). Initial studies applying the concept in Europe similarly stressed the heritage value of traditional biocultural diversity and the importance of conserving traditional European landscapes that testify how people have interacted with nature (Agnoletti and Rotherham 2015). This heritage and conservation focus has been criticized for failing to recognize that culture is dynamic (Cocks 2006). Studies in both tropical countries (Cocks *et al.* 2011; Cocks and Wiersum 2014) and Europe (Elands and Van Koppen 2012; Elands *et al.* 2015) have shown that the dynamics of biocultural diversity not only involve loss of historically created biocultural diversity due to modernization, but also involve the adjustment of cultural interactions with biodiversity in response to changing socioeconomic conditions and human creativity (see also Turnhout *et al.* 2013). Buizer *et al.* (2016) argue that the concept of biocultural diversity should not be interpreted as a definitive concept that is prescriptive of what to see (e.g., endangered biocultural diversity amongst indigenous and traditional societies), but rather as a sensitizing and reflexive concept suggesting directions along which to look in dealing with the diversity and dynamics in human-nature interactions.

In this paper I use the concept of biocultural diversity as an analytical device to assess the new interest in wild forest products in Europe as a process of biocultural dynamics. I first elaborate the nature of wild forest products as a biocultural phenomenon. These products are often characterized as deriving from species that have not been impacted by the process of domestication. Alternatively, they may be conceived as deriving from domesticated species that have been reintroduced into the wild. I characterize the two processes of domestication and rewilding as a manifestation of biocultural dynamics involving biological, environmental, and cultural dimensions. Next, I illustrate the diversity in wild forest products in Europe through a comparative review of two types of wild forest products in the Netherlands: wild meat and wild food plants. I conclude that the development of wild forest products involves both a return to earlier phases of domestication in a biological sense and a new phase in the process of acculturation of forest products to the human domain.

## Wild Forest Products as a Biocultural Phenomenon

Even though the term ‘wild forest products’ is increasingly used in Europe (Wolfslehner *et al.* forthcoming), the precise meaning is open to different interpretations. According to the dictionary the adjective ‘wild’ may refer not only to untamed objects that exist autonomously from human impact, but also to objects that are not domesticated by humans, or to objects that do not adhere to human social or cultural norms. Whereas the first interpretation is biologically based, the other two emphasize human practices and norms and are thus culturally oriented. On the basis of these different meanings wild forest products may be conceived of as products that are derived from wilderness areas, or from traditional nature-analogue production systems, or from species that are not acculturated in the sense of being subject to institutionalized practices. In several policy documents the notion of wild products is interpreted as referring to products that are derived from wild species that occur in self-maintaining populations in natural or semi-natural ecosystems and can exist independently from human action (Heywood 1999). This is reflected in EU Regulation 2092/91 on organic production and labelling that defines the collection of wild products as concerning the collection of edible plants or parts thereof that are growing naturally in natural areas, forests, and agricultural areas. The World Health Organization (WHO), the International Union for the Conservation of Nature (IUCN), and the World Wildlife Fund (WWF) adhere to an International standard for sustainable collection of wild medicinal and aromatic plants that refers to the practice as the gathering a non-cultivated native or naturalized resource from its natural habitat (Censkowsky *et al.* 2007). Thus, wild products are generally considered as deriving from autonomously growing plants that may occur not only in undisturbed wilderness areas, but also in human adapted environments such as managed forests and even agricultural areas.

From this perspective, wild plants are characterized in contrast to domesticated plants that have been subject to purposeful selection and cultivation (Leakey and Newton 1994). This reflects the long-standing distinction in Western thought between nature and culture (Harris 1996). Nevertheless, this distinction is problematic (Harris 1996). It is not always clear whether a product may be regarded as harvested from a spontaneously growing species or a species that has been affected by human practices. For instance, some products that are collected in the natural environment may still be subject to manipulation by collectors (e.g., by removal of competing species or stimulating regeneration), while products growing wild in one country may actively be managed in another (Censkowsky *et al.* 2007; Wolfslehner *et al.* forthcoming). Thus, rather than interpreting wild and domesticated as two contrasting states of being, it may be preferable to consider wild and domesticated species as representing outcomes of a dynamic process of people-nature interactions, as proposed by Harris (1989, 1996). Recently, the process of re-wilding was introduced to characterize new approaches to recreate more naturalistic environments by stimulating autonomous ecological processes in degraded environments (Jorgensen 2015; Lorimer *et al.* 2015; Van Maanen and Convery 2016). The new interest in wild forest products in Europe may therefore be conceived of as a new phase of biocultural dynamics involving the addition of the process of re-wilding to the long history of domestication. In order to understand how the understanding of wild forest products relates to biocultural dynamics it is useful to consider the nature of the processes of domestication and re-wilding in more detail.

### Domestication

The differentiation between domestication as a way of being and as a dynamic process reflects the long history of the concept among various scientific disciplines (Harris 1989, 1996). The concept can be interpreted in different ways (Harris 1989; Wiersum 2008). Some scientists define it in a biological sense as referring to the process of gradual adaptation of a species’ morphological and genetic characteristics to specific uses and man-made growing environments (Leakey and Newton 1994) whereby a species is considered domesticated when it has purposefully been selected for specific genetic characteristics and when it is propagated and cultivated in specialized production systems. Others consider that domestication also includes a process of acculturation to the human domain where nature and culture interact (Harris 1989; Chase 1989; Hladik *et al.* 1993). In such a comprehensive sense domestication is considered a multi-facetted process of biocultural dynamics in which a progressively closer interaction between people and biological resources takes place. In this process three dimensions may be distinguished (Harris 1989; Wiersum 1997, 2008):

- Modification of a species’ biological characteristics by developing high yielding phenotypes and cultivars;
- Domestication of a species’ biophysical environment by developing adapted environments in the form of agrarian production systems such as plantations, orchards, and kitchen gardens;
- Acculturation of a species to the human domain by developing cultural practices for using biodiversity for both experiential and material purposes (Posey 1999). During this process the institutional arrangements in respect to access to resources (e.g., in the form of land tenure arrangements) and to markets gradually become more complex (Laird *et al.* 2010; Wiersum *et al.* 2014).

Due to the multidimensional nature of the process of domestication, this process may evolve in several stages. Ethnobotanical studies in tropical countries (Harris 1989; Wiersum 1997) have illustrated the following: (1) free procurement of wild products, (2) socially controlled procurement of wild products, (3) stimulated production of species by modifying their environment and phenotype, (4) cultivation of sometimes selected genotypes and/or modified phenotypes in man-made environments, (5) cultivation of genetically adapted cultivars in man-made environments. The interpretation of domestication as denoting a state of being does not reflect this process of gradual change from gathering ‘untamed’ species to cultivation of selected cultivars in man-made agrarian environments. Consequently, the interpretation of the new interest in wild forest products as a renewed appreciation of original forest species that were historically taken out of the forest to be cultivated in specialized horticultural or animal husbandry production systems should be considered as a first approximation only.

### Re-Wilding

Whereas the concept of domestication refers to the process of gradual intensification in people – nature interactions and artificialization of production systems, the concept of re-wilding refers to the de-intensification of these interactions and the reintroduction of more naturalness in ecologically degraded areas (Jorgensen 2015; Lorimer *et al.* 2015; Van Maanen and Convery 2016). The concept emerged in the USA and Europe in response to growing concerns on the negative ecological impacts of agricultural production systems on rural landscapes. Originally the focus was on the re-creation of wilderness areas by securing large and well-connected core protected areas and releasing key-stone carnivorous species. Subsequently it became further modified as also concerning the abandonment of productive lands and the reintroduction of more wildness in ecologically impoverished areas by stimulating natural ecological processes. The various approaches to re-wilding have given specific attention to the role of animal species at the higher trophic levels in regulating ecological processes. Gradually several approaches to re-wilding became recognized (Jorgensen 2015; Lorimer *et al.* 2015; Van Maanen and Convery 2016):

- Re-wilding through pleistocene mega-fauna replacement or taxon replacement on islands;
- ‘Top-down’ re-wilding through reintroduction of carnivorous key stone species operating at the top of the ecological pyramid;
- ‘Bottom-up’ re-wilding through reintroduction of naturalistic grazing by more or less wild animals.

Whereas the approaches of pleistocene mega-fauna replacement and ‘top-down’ re-wilding aim at returning lands to an ‘untamed’ state characterised by the presence of the original fauna and/or key-stone species and prevalence of autonomous ecological processes, the stimulation of ‘bottom-up’ re-wilding involves the reintroduction of more wildness in half-natural systems by stimulating more naturalistic forms of grazing. These different approaches may be based either on the stimulation of natural autonomy through enhancement of natural processes that were impaired by humans or on the improvement of the ecological functioning through the enhancement of naturalistic processes that positively impact the ecological integrity of the area (Arts *et al.* 2016). However, it does not involve a return to a pre-human state in the sense of excluding people from nature (Hall 2014). Rather, it reflects new forms of environmental consciousness and new forms of experiencing and living with nature through the provision of new opportunities to interact with nature (Arts *et al.* 2016). Thus, similar to the concept of domestication, re-wilding reflects a multi-facetted process of bio-cultural dynamics involving both ecological and cultural relations.

## New Interest in Wild Forest Products in the Netherlands

The notion that the new interest in Europe in wild forest products reflects a new phase in bio-cultural dynamics may be illustrated by two examples of the re-wilding of forest products in the Netherlands. In the nineteenth century in this country forest and related wildlands (as uncultivated land was called at that time) still played an important role in producing a variety of NWFPs. These lands were used for grazing livestock, provided industrial resources in the form of oak bark used in the tanning industry as well as forest foods such as berries, nuts, and game for either household or commercial purposes. However, in the twentieth century a clear differentiation between agriculture and forestry emerged with agriculture being focused on specialized agrarian production and forestry as a combination of timber production, environmental conservation, and provision of amenity services (Schmidt *et al.* 1999). Most forest foods had become domesticated and were produced in a specialised horticultural sector. In addition, hunting became less popular and manufactured chemical products replaced industrial uses of NWFPs. According to European statistics on forest use, only Christmas trees and game are at present formally recognized as NWFPs from the Dutch forests (Forest Europe, UNECE and FAO 2011). These data, however, do not reflect the fact that collection and use of wild forest products, for instance as part of recreational activities, still prevails (Beugelsdijk 2006). Similar to other European countries, recent interest in wild forest products is growing in the Netherlands in response to new consumer interests in actively experiencing the natural and cultural identity of forest products. This is reflected in the emergence of two categories of wild forest production, i.e., (1) wild meat production from naturalistic grazing systems, and (2) collection of forest food plants. The development of these two categories of wild forest products reflects different pathways of biocultural dynamics.

### Wild Meat Production from Naturalistic Grazing Systems

The increasing interest in ‘wild’ faunal products from naturalistic grazing emerged as a result of new professional approaches to the management of forest and nature areas in the Netherlands (Lorimer *et al.* 2015). The introduction of naturalistic grazing aimed to strengthen the ecological processes of habitat differentiation and seed dispersal in these areas. In order to stimulate such autonomous ecological processes, initially back-bred domesticated livestock such as Scottish Highlands and Heck cattle and Exmoor ponies that are considered as reflecting wilderness species were introduced in conservation and forest areas (Lorimer *et al.* 2015; Van Maanen and Convery 2016). This ecological approach has been complemented by a heritage-oriented approach involving the reintroduction of ancient livestock varieties such as heath cows, regional breeds of sheep, and forest pigs. The historic use of these livestock breeds has resulted in half-natural landscapes that are at present highly valued for reflecting regional landscape identity. Consequently, the reintroduction of historic breeds involves the stimulation of ancient agrobiodiversity. Notwithstanding their less domesticated status in a biological sense, both the ‘re-naturalized’ animals and the ancient livestock breeds are still ‘civilized’ in the sense of being subject to several institutional arrangements. Rather than legally being classified as wild species, they are still considered as livestock species subject to animal welfare and health control regulations. Their re-introduction has resulted in intensive discussions between conservationists and animal welfare proponents about their management (Klaver *et al.* 2002). One very contentious issue is whether dead animals should be left in the re-wilded areas to naturally decompose. A more common practice is to cull animals to prevent overgrazing by the herds that have gradually grown in size due to the increasing popularity of naturalistic grazing. The meat of the culled animals is increasingly marketed as a nature or wilderness product in specialised niche markets, e.g., in the form of sale to members of nature conservation organisations or of marketing through specialised cooperatives. Moreover, the reintroduction of the animals is often heralded as increasing the recreational value of forest and nature areas. Considering the experiential values of the naturalistic grazers and the wild meat products and the new forms of institutionalisation of wild meat production, the wild meat products can be considered as highly domesticated in the sense of being acculturated to the present human environment.

### Collection of Naturalistic Forest Plant Products

In contrast to the wild meat products, the new interest in collecting ‘wild’ forest food plants did not primarily emerge in response to the new interest in re-wilding ecologically degraded areas, but rather in response to growing consumer demands for natural health products as well as for actively experiencing forests through gathering and tasting wild forest products. These new cultural interests are reflected in the publication of quickly growing number of recipe books on natural foods, the introduction of food walks and food collection events in forest/nature areas, as well as organisation of wild food related leisure activities such as forest food fairs and courses on wild food preparation. The appeal of gathering wild foods is heralded as "a style of living in which you get to know your environment and nature as well as a sport and a search trip that points to the future" (Maijer 2015). Many of these novel activities have been initiated by local entrepreneurs and/or volunteer organisations rather than by professional management organisations. Increasingly, professional nature and forest management organisations also provide new opportunities for food plant collection by developing new types of food gathering forests.^1^ These forests are enriched by natural fruit-producing species and/or by old varieties of cultivated fruit species. From a biological point of view, these forests involve a return to earlier phases of domestication of forest food plants, but from an acculturation point of view they are still domesticated in respect to species selection and management organisation. Their instututionalization is reflected in their management arrangements  involving active participation by community and non-governmental organisations. It is also illustrated by their emergence in mostly peri-urban areas within the context of urban transition programmes aiming at the stimulation of nature services for urban populations.

### Comparative Bio-Cultural Features

The main characteristics of the two approaches to wild forest production in respect of biological, environmental and cultural (and related institutional) characteristics are summarized in Table 1.

**Table 1:** Main characteristics of two categories of wild forest products in the Netherlands

Production system	Biological characteristics	Characteristics of production areas	Main cultural values involved	Institutional characteristics
Meat production from naturalistic grazing	Back-bred forefathers of livestock such as Scottish Highlands, Heck cattle and Exmoor pony reflecting traditional wilderness species	Conservation areas in which autonomous ecological processes are stimulated. Wild meat as by-product from population management to prevent overstocking	Ecologically-oriented values in respect of stimulating natural authenticity and autonomy as well as improved ecological functioning	Management by nature conservation organisation with the aim to improve ecological processes. Marketing by niche market organisations
	Ancient livestock varieties such as heath cows, regional breeds of sheep and forest pigs	Traditional half-natural livestock grazing environments	Combination of ecologically oriented values in respect to stimulating improved ecological functioning and cultural heritage values in respect of conserving traditional agrobiodiversity	Management by joint nature and heritage conservation oriented organisations Marketing by niche market organisations
Collection of forest food plants	Spontaneously growing food plants	Appropriate forest ecological niches	Experiential values in respect of actively interacting with nature by gathering wild food plants and tasting nature	Self collection by consumers stimulated by civil society organisations propagating use of nature food and health products
	Traditional varieties of historically horticulturalized species	Often peri-urban forest areas that have been enriched by fruit and nut producing species	Experiential values in respect of actively interacting with nature by being involved in the collaborative production of forest foods from non-artificialized environments	Community-based and collaborative management arrangements

The new interest in wild forest products involves two main types of bio-cultural dynamics. Firstly, they reflect new interests in the development of more vital natural ecological processes in degraded forested landscapes through the reintroduction of either rewilded breeds of animals or ancient livestock breeds. In the management of such areas, the focus on the re-introduction of more naturalistic forest ecological processes was gradually extended to the use of the provisional ecosystem services they provide. Consequently, products of nature-oriented management systems are increasingly sold as nature or wilderness products in niche product markets. Secondly, the new interest in wild forest products is related to new interests in the cultural services of forests in respect of providing identity and heritage values as well as recreational experiences. The new forms of experiencing nature stimulated not only increased use of original biodiversityc for consumption or sale, but also increased interest in the reintroduction of varieties from the early stages of domestication in new types of recreational forests.

## Discussion and Conclusion

The interest in NWFPs in tropical countries originally focused on wild products that were collected from more or less spontaneously growing species in natural or semi-natural forests. The characteristics of these wild products conform the formal definitions of wild products as formulated by various international organisations (Heywood 1999; Censkowsky *et al.* 2007). However, in Europe wild forest products often do not conform to these characteristics. As illustrated by the examples from the Netherlands, similar to the stimulation of re-wilding (Arts *et al.* 2016), the recent interest in wild forest products in Europe involves not merely a resurgence of interest in forest products from ‘wild’ species growing in natural environments, but also new interest in natural production that resembles earlier stages of the multifaceted process of domestication.

However, interest in wild forest products does not just reflect a renewed appreciation of more natural ecological conditions and less artificialized forest production systems. As illustrated by its relation to new cultural interests in recreation, local landscape identity, and culinary enjoyment, it also reflects an increased emphasis on wellbeing rather than basic livelihood concerns. As also argued in respect of re-wilding (Arts *et al.* 2016), from a socio-cultural perspective the wild products cannot be considered as deriving from outside the human domain in the sense of not being subject to human norms. Indeed, considering the great variety of cultural practices in using natural products in tropical countries, the interpretation of wild products as not reflecting human norms is not correct. Rather, the use of NWFPs reflects various types of bio-cultural interaction (Posey 1999; Persic and Martin 2008; Pretty *et al.* 2009). Although several forms of traditional bio-cultural interactions may be lost due to modernization (Pilgrim *et al.* 2008; Pilgrim and Pretty 2010; Rapport and Maffi 2010), others may endure (Cocks *et al.* 2011; Cocks and Wiersum 2014). And as illustrated by the Netherlands examples, new types of biocultural interactions, e.g., re-wilding forest production, may emerge (Elands and Van Koppen 2012).

The examples from the Netherlands demonstrate how such new interest in wild forest products may involve several approaches to re-wilding forest production. On the one hand, it may be related to the growing appreciation of autonomous ecological processes and related ecological services, reflected not only in the growing interest in collecting products from spontaneously growing forest species, but also in the reintroduction of different kinds of key-stone species through re-wilding programmes (Lorimer *et al.* 2015; Van Maanen and Convery 2016). Although these programmes are primarily focused on making better use of the ecological regulation services of the reintroduced species, they have resulted in an increased appreciation of their provisioning services. Alternatively, the new interests in wild forest products may also be related to the growing interest in the cultural heritage values of historic forest production systems (Agnoletti and Rotherham 2015). This is reflected in the reintroduction of ancient varieties of both botanical and faunal species in forests.

Although these two approaches to re-wilding forest production differ in respect of their ecological orientation, they are both embedded in new cultural orientations regarding the significance of forests. These are increasingly appreciated because they provide not only ecological services, but also cultural services in respect to landscape identity, recreation, and self-fulfilment, which has resulted in a change from the traditional commodity-oriented focus on forest production to a more service-oriented focus involving increased attention to the experiential values of forests (Mather 2001). The new forms of experiencing forests are related to a socioeconomic transition in Europe involving increased rates of urbanisation and a change from a production and manufacturing economy to a service and knowledge economy (Mather 2001; Konijnendijk 2003; Hoogstra *et al.* 2004). Consequently, new interest in wild forest products often emerges in peri-urban areas rather than remote rural areas. This change requires the development of new institutional arrangements for the production and marketing of the wild forest products, e.g., in the form of new types of participatory management systems, new types of niche marketing systems, and linking of the use of wild forest product to the recreational and leisure sectors (Pettenella *et al.* 2007; Wolfslehner *et al.* forthcoming). As a result of such institutional innovations, the wild forest products are often subject to complex governance arrangements (Laird *et al.* 2010). Dealing with this institutional complexity requires new forms of forest-related entrepreneurship (Niskanen 2008; Elands *et al.* 2015; Ludvig *et al.* 2016). As seen in the Netherlands examples, whereas the return to naturalistic conditions is often spearheaded by professional organisations, the crafting of new heritage-inspired production and marketing systems is often initiated by civil society organisations. Thus, the new interest in wild forest products involves the creation of a variety of novel bio-cultural interactions that reflect diversity in experiencing forests by either professionals or local people. Such a duality in living with bio-cultural diversity has recently also been noted in respect to the management of urban green spaces (Elands *et al.* 2015). Thus, from a bio-cultural perspective the new interests in wild forest products can be considered as involving a new phase in the human acculturation of forests in modern peri-urbanized areas. The interest in wild forest products reflects not just a renewed appreciation of more natural ecological conditions and less artificialized forest production systems, but also new interests in making better use of the natural and cultural heritage values of forests and in developing novel approaches to experiencing nature and living with biodiversity.

## Footnotes

1 In addition to gathering forests, food forests are also being developed in the form of multi-storeyed cultivation systems. Although these forest analogue cropping systems involve a naturalistic production system, they involve a manipulation of the biophysical environment by the creation of an adapted agrarian system.
